# miR-23b and miR-27b are oncogenic microRNAs in breast cancer: evidence from a CRISPR/Cas9 deletion study

**DOI:** 10.1186/s12885-019-5839-2

**Published:** 2019-06-28

**Authors:** Bethany N. Hannafon, Angela Cai, Cameron L. Calloway, Yi-Fan Xu, Roy Zhang, Kar-Ming Fung, Wei-Qun Ding

**Affiliations:** Department of Pathology, University of Oklahoma Health Sciences Center, 940 Stanton L. Young Blvd., BMSB401A, Oklahoma City, OK 73104 USA

**Keywords:** CRISPR/Cas9, microRNA, Breast cancer

## Abstract

**Background:**

Altered expression of microRNAs (miRNAs) is known to contribute to cancer progression. miR-23b and miR-27b, encoded within the same miRNA cluster, are reported to have both tumor suppressive and oncogenic activity across human cancers, including breast cancer.

**Methods:**

To clarify this dichotomous role in breast cancer, miR-23b and miR-27b were knocked out using CRISPR/Cas9 gene knockout technology, and the role of endogenous miR-23b and miR-27b was examined in a breast cancer model system in vitro and in vivo.

**Results:**

Characterization of the knockout cells in vitro demonstrated that miR-23b and miR-27b are indeed oncogenic miRNAs in MCF7 breast cancer cells. miR-23b and miR-27b knockout reduced tumor growth in xenograft nude mice fed a standard diet, supporting their oncogenic role in vivo. However, when xenograft mice were provided a fish-oil diet, miR-27b depletion, but not miR-23b depletion, compromised fish-oil-induced suppression of xenograft growth, indicating a context-dependent nature of miR-27b oncogenic activity.

**Conclusions:**

Our results demonstrate that miR-23b and miR-27b are primarily oncogenic in MCF7 breast cancer cells and that miR-27b may have tumor suppressive activity under certain circumstances.

## Background

miRNAs are small, non-protein coding RNAs, ranging in sizes from 17- to 25 nucleotides that regulate mRNA expression at the post-transcriptional level by complementary binding to corresponding sequences in the 3′ untranslated region (3’UTR). miRNAs are predicted to regulate > 90% of protein-coding genes, thereby making them the largest class of gene regulators [1]. Due to their broad regulatory role and their various target mRNAs, miRNAs can regulate various normal and pathogenic cellular processes including cell cycle, tumorigenesis, migration/invasion, and angiogenesis thus functioning as oncogenes or tumor suppressors [2].

The miR-23-27-24 family consists of two paralogs with the miR-23a cluster (miR-23a-27a-24-2) found on chromosome 19 and the intragenic miR-23b cluster (miR-23b-27b-24-1) located on chromosome 9 within the C9orf3 gene [3]. miR-23a and miR-27a differ from their paralogs by only one nucleotide near their 3′ end, however, diverse expression patterns are observed among these two clusters [4] and both are predicted to have largely identical targets, although less than 15% of the same targets have been validated according to miRTarBase [5].

Based on the current literature, miR-23b and miR-27b have a dichotomous role in cancer progression. miR-23b expression is down-regulated in human glioma, prostate, bladder, breast, and gastrointestinal cancer, and has been shown to suppress tumor growth, invasion, angiogenesis, and metastasis, and influence chemo-resistance and tumor cell dormancy [6–15]. While other studies have shown that expression of miR-23b is elevated in various cancers and may also function as an oncogene by promoting tumor growth, proliferation, and metastasis in glioma, prostate, breast, and gastric cancers and is associated with poor progression-free survival in ovarian cancer [16–20]. Likewise, expression of miR-27b is increased in glioma, lymphoma, cervical, breast cancer, and acts as an oncogene by promoting proliferation, inhibiting apoptosis, and inducing migration and invasion [18, 21–23]. While other studies have shown that expression of miR-27b is reduced in lung, prostate, colorectal, gastric, and bladder cancer and it acts as a tumor suppressor by limiting proliferation, inhibiting tumor progression and angiogenesis, and the loss of miR-27b expression promotes epithelial to mesenchymal transition and breast cancer metastasis [9, 24–28]. Also, miR-23b and miR-27b have been shown to both promote [29, 30] and repress angiogenesis [31–33].

Studies examining the role of miR-23b and miR-27b in breast cancer have also demonstrated contrasting results, oncogenic or tumor suppressive. Several groups have demonstrated the cancer-promoting role of miR-23b and miR-27b in breast cancer. For example, Jin et al., showed that inhibition of miR-23b and miR-27b by stable expression of anti-miR-23b/27b constructs reduces cell proliferation, anchorage-independent growth, migration, and invasion by metastatic breast cancer cell lines (MDA-MB-231-4175) in vitro and reduced tumor growth and metastases in vivo, in part by negatively regulating the target tumor suppressor gene, Nichscharin [18]. Likewise, Ell et al. demonstrated that miR-23b/27b expression is increased in three isogenic breast cancer progression cell line series (mouse 4 T1 series, and human MCF10A and MDA-MB-231 series). In this study, overexpression of the miR-23b-miR-27b-24 cluster in weakly metastatic 4TO7 cells led to an increase in the number of metastatic lung lesions in vivo in a tail-vein injection mouse model [34]. Wang et al. showed that inhibition of miR-27b using antagomirs in highly invasive breast cancer cells (MDA-MB-231-4175) suppresses cell invasion via upregulation of suppressor of tumorigenicity 14 (ST14), meanwhile, miR-27b overexpression stimulated invasion in moderately invasive breast cancer cells (ZR751) [23]. In contrast, other studies have shown a cancer-inhibitory effect of miR-23b and miR-27b in breast cancer. In a study by Pellegrino et al., miR-23b inhibition using a miRNA sponge construct leads to an increase in cell migration and metastatic spread in vivo, by directly inhibiting transcripts involved in cytoskeletal remodeling and motility in metastatic breast cancer cells (MDA-MB-231) [10]. Another study showed that down-regulation of miR-27b could confer tamoxifen resistance through up-regulation of NR5A2 and CREB1 expression in breast cancer cell lines (MCF7 and T47D), while upregulation of miR-27b expression enhanced the sensitivity of breast cancer cells to tamoxifen, suggesting a role for miR-27b in hormone therapy for breast cancer patients [35].

The observations that miR-23b and miR-27b act as either oncogenes or tumor suppressors in various cancer models are possibly due to the use of different model systems among various studies, but also likely due to the fact that these previous studies are unable to completely eliminate expression of miR-23b or miR-27b to elucidate their functional role in cancer. Because of their potential relevance in the development of anti-cancer therapeutics [36], we sought to determine the specific role of miR-23b and miR-27b in breast cancer progression by genetic depletion of these miRNAs using CRISPR/Cas9 engineering technology. We developed miR-23b and miR-27b null breast cancer cell lines and characterized their phenotype in vitro using various phenotypic assays and in vivo using a xenograft mouse model. Our results indicate that miR-23b and miR-27b are primarily oncogenic in breast cancer cells.

## Methods

### Cell culture

We purchased the human breast cancer cell line MCF7 from the American Type Culture Collection (Manassas, VA). Upon receipt of the cells, low passage number aliquots were prepared and cryopreserved, and all experiments were conducted with low passage number cells. We have regularly checked the line for mycoplasma contamination by a PCR-based method and only mycoplasma free cells were used for this study. The cell line was genetically authenticated to be MCF7 by STR analysis at the Arizona University Genetic Core in 2018. The cells were cultured in DMEM containing 10% fetal bovine serum (FBS), 100 IU/ml penicillin and 100 μg/ml streptomycin (Corning/ Mediatech, Inc. Manassas, VA), at 37 °C, 5% CO_2_ in a humid environment. Media supplemented with 10% FBS contains approximately 0.44% n-3 polyunsaturated fatty acids [37] and that total free fatty acid concentration in the fetal calf serum is around 200 μM, of which DHA account for about 3.9% (below 10 μM) [38].

### Single guide RNA design and lentiviral transduction

The sgRNAs were designed as recently described [39]. The DNA sequence corresponding to the annotated stem-loop miRNA (miRBase) was used as an input sequence. The guides with the highest scores and/or closest proximity to the mature miRNA sequence were chosen. Oligonucleotides complimentary to the selected sgRNA sequence were synthesized with *BsmBI* overhangs (Integrated DNA Technologies). The oligonucleotides were ligated into the lentiCRISPR v2, a gift from Feng Zhang (Addgene, # 52961) [40]. miR-23b-sgRNA-1: 5′-CGTGGTTGCGTGGTAATCCC-3′; miR-23b-sgRNA-2: 5′-GCTCTGGCTGCTTGGGTTCC-3′; miR-27b-sgRNA-1: 5′-GGTTTCCGCTTTGTTCACAG-3′; and miR-27b-sgRNA-2: 5′-AGGTGCAGAGCTTAGCTGAT-3′. Lentiviral particles were produced as previously described [32]. The MCF7 cells were infected with lentivirus in the presence of 8 μg/ml polybrene (Sigma-Aldrich). Approximately 48 h post-infection, the cells were selected by treating with 1 μg/ml puromycin (InvivoGen, San Diego, CA) for 3 days.

### Genomic PCR, T7 endonuclease assay, and sequencing

We extracted genomic DNA from wildtype and Cas9/sgRNA transduced and puromycin selected MCF7 cells using the Pure Link Genomic DNA Mini-kit (Invitrogen). Primers were designed to amplify a ~ 700 base fragment surrounding the sgRNA cleavage site. miR-23b primers: forward 5′- CTCCCCAGCATCTTCGATCC-3′ and reverse 5′- GAGGTCATCGCTGGGCATAA-3′; and miR-27b primers: forward 5′- CAGGTGCATCTCGTAGCTCT-3′ and reverse 5′- TTTGCTCAAGGGCTCGACTC-3′. The genomic loci of interest were amplified by PCR using Phusion High-Fidelity DNA Polymerase (Thermo-Scientific). We applied the T7 Endonuclease assay to evaluate the gene editing efficiency [39]. Single cell clones of each transduced cell line were expanded. The PCR amplicons of each clone were inserted and replicated in the pCR™4-TOPO® TA vector (Thermo-Fisher), and sent for Sanger sequencing using the T7 primer (5′ TAATACGACTCACTATAGGG 3′).

### RNA extraction and real-time PCR

Total RNA was prepared with TRIzol reagent (Invitrogen/Thermo-Fisher) followed by the PureLink RNA Mini Kit (Invitrogen). RNA concentration was determined on the NanoDrop ND-100 Spectrophotometer (NanoDrop Technologies). For miRNA expression analysis, cDNA was synthesized from 100 ng of total RNA using the Quanta qScript microRNA cDNA Synthesis Kit (Quanta Biosciences). The cDNA was combined with 2X iTaq Universal SYBR Green Mix (Bio-Rad), 200 nM PerfeCTa Universal PCR primer, and 200 nM PerfeCTa microRNA assay primers for miR-23a-3p, miR-23b-3p, miR-27a-3p, miR-27b-3p, and miR-24b-3p, and the normalization control SNORD44. For mRNA expression analysis, cDNA was synthesized from 500 ng total RNA using the iScript cDNA Synthesis Kit (Bio-Rad). The cDNA was diluted (1, 10) and combined with 500 nM of forward and reverse primers. Primer sequences are as follows: AMOTL1, forward 5′-CGAGGGACTGAACTAGCCAT-3′ and reverse 5′-AGGGGACCCTTTCACCG-3′; VHL, forward 5′-ACGGACAGCCTATTTTTGCCA-3′ and reverse 5′-TCTTCGTAGAGCGACCTGAC-3′; ST14, forward 5′-CACCCAACATTGACTGCACAT-3′ and reverse 5′-GCAGTATTTCTCCCCGTTGAT-3′; APC, forward 5′-AACGAGCACAGAGGTCATC-3′ and reverse 5′-GGCTGTTTCATGGTCCATTCG-3′; and the normalization control 36B4, forward 5′-ATCAACGGGTACAAACGAGTCCTG-3′ and reverse 5′- AAGGCAGATGGATCAGCCAAGAAG-3′. PCR reactions were run on the Bio-Rad CFX 96 Real-Time PCR (Bio-Rad, Hercules, CA) as described [39]. Relative miRNA expression was assessed using the differences in normalized Ct (ΔΔCt method) after normalization.

### MTS cell proliferation assay

Cells were plated onto a 96-well plate at a density of 12,000 cells/well in quadruplicate, and cultured at 37 °C with 5% CO^2^ for 1–3 days. For each well, the attached cells were incubated in 100 μL growth medium supplemented with 20 μL CellTiter 96® AQueous One Solution (Promega, Madison, WI, USA) and incubated for 1 h. The absorbance value at 495 nm was recorded using a spectrometer.

### Wound healing/migration assay

To assess cell migration 1 × 10^6^ cells were seeded to a 6-well plate in triplicate. When the cells reached at least 90% confluency a sterile 200 μl micropipette tip was used to make 3–4 separate “wounds” through the cells. Culture medium and any floating cells were removed and replaced with fresh medium. Cells were incubated at 37 °C with 5% CO_2_. Initial wound images and the width of each “wound” were measured at the same position for the next 3 days on an Olympus IX51 inverted microscope using the cellSens Standard imaging software (Olympus, Tokyo, Japan).

### Soft agar colony formation assay

The soft agar colony formation assay was used to assess the anchorage-independent growth of the miRNA knockout cells. For the bottom layer, sterilized 1% agarose LE was diluted 1:1 in complete cell culture medium and 1 ml was transferred to each well of a 6-well plate, for a final agarose concentration of 0.5%. The agarose was allowed to solidify at room temperature for 30 min. To prepare the upper layer, cells were harvested and diluted to a density of 10,000 cells/ 0.5 mL of complete medium. To this cell suspension, 0.5 ml of sterilized 0.6% agarose was added, and 1 ml of the mixture was transferred to each well, for a final agarose concentration of 0.3%. The cell/agarose mixture was allowed to solidify at room temperature for 30 min. The cells were incubated at 37 °C with 5% CO_2_ for 20–30 days. Every 2–3 days 0.2 ml of complete medium was added to each well to prevent drying. Colonies were stained with 0.005% crystal violet in 25% methanol for 1 h. Images and colony counts were obtained using the Optronix Gel Count (Oxford Optronix Ltd.).

### Clonogenic assay

To assess the ability of the miRNA knockout cells to proliferate indefinitely, the cells were seeded at a density of 500 cells/well in a 6-well plate and incubated at 37 °C with 5% CO_2_ for 10–14 days. The culture media was refreshed every 3–4 days. Cells were stained with 0.5% crystal violet in 25% methanol for 15 min. The stained cells were washed 3 times with tap water and allowed to dry on the bench. Images and colony counts were obtained using the Optronix Gel Count (Oxford Optronix Ltd.).

### Human breast cancer xenograft mice

Five-week-old female athymic nude mice (NCRNU-F, Taconic Farms, Inc.) were used for the in vivo study. The animal research protocol was approved by and performed per the policies and guidelines of the University of Oklahoma Health Sciences Center Institutional Animal Care and Use Committee (Institute IACUC Protocol #100861–14-025-SSH). Mice were kept in a conventional animal facility, with a 12-h on-off light cycle in standard rectangular mouse cages, with 5 mice per cage. Cages were lined with adsorbent bedding and enriched with crinkle paper for nesting. Mice had continuous access to water and food. One week prior to breast cancer cell inoculation the mice were randomly divided into two groups, a standard diet consisting of 0.45% n-3 fatty acids (PicoLab Rodent Diet-20 #5053, LabDiet), a fish oil diet consisting of 7.5% n-3 fatty acids (Envigo TD.110647), and fed with the assigned diet during the whole duration of the experiment. Three days before cell inoculation the mice were supplemented with 17β estradiol in the form of a 0.36 mg/pellet, 60-day release (SE-121, Innovative Research of America) implanted subcutaneously in the intrascapular region. The MCF7 miR-23b and miR-27b knockout or control cells were inoculated at a density of 5 × 10^6^ in 100 μl PBS containing 5 μg/ml Matrigel (BD Biosciences) subcutaneously into the mammary fat pad (*n* = 6 mice per group). Mouse weight and tumor size measurements were initiated 7 days post inoculation and monitored three times per week. Tumor measurements were taken using digital calipers. Tumor volume (v) was measured using the following formula: v = (l × w^2^) × 0.5, where l is the length and w is the width of the tumor measured in mm. Tumor weight (W, in grams) was estimated by the following formula: W (g) = v (mm^3^) × 0.001. When the estimated tumor weight reached or exceeded 10% of the body weight, the mouse was euthanized by CO_2_ asphyxiation. No adverse events were observed. Tumor tissues were dissected, fixed in 10% buffered formalin phosphate, followed by paraffin embedding, sectioning, hematoxylin, and eosin (H/E) staining. Survival analysis was conducted in GraphPad Prism (version 7). The date of death was entered for mice within each group over the entire study period. The survival curves were compared using the Log-rank test (Mantel-Cox).

### Immunohistochemical staining of CD31 and Ki67

Tumor tissues were dissected from mice upon sacrifice and fixed in a 10% neutral buffered-Formalin solution and embedded in paraffin wax. FFPE tissues were sectioned (4 μm), mounted on positively charged slides and dried overnight at room temperature. Slides were then incubated at 60 °C for 45 min, followed by deparaffinization, and rehydration in an automated Multistainer (Leica ST5020). Slides were transferred to the Leica Bond-III™ and treated for target retrieval at 100 °C for 20 min in a retrieval solution (at pH 6.0 or pH 9.0). Endogenous peroxidase enzyme was blocked with a peroxidase-blocking reagent, followed by primary antibody incubation for 60 min. Tumor sections were evaluated by immunohistochemistry for neovascularization using an antibody against CD31 (1:50; ab28364, Abcam) and for proliferation using an antibody against Ki67 (1:800; ab15580, Abcam). Post-primary IgG-linker and/or Poly-HRP IgG secondary antibody reagents were used for detection with 3, 3′-diaminobenzidine tetrahydrochloride (DAB) as chromogen. Slides were counterstained with hematoxylin. Stained slides were dehydrated (Leica ST5020) and mounted (Leica MM24). Primary antibody specific positive and negative controls (omission of primary antibody) were stained in parallel. Stained slides were scanned and analyzed using the Aperio CS2 scanner and ImageScope software v12 (Leica Biosystems). To analyze nuclear Ki67 staining the Nuclear Staining Algorithm was used to calculate the percent of nuclei positive among 4 randomly chosen areas within each xenograft tumor section. The Microvessel Analysis Algorithm was used to quantify the number of CD31 positive microvessels present on each tumor xenograft section.

## Results

### CRISPR/Cas9 knockout of miR-23b and miR-27b in MCF7 cells

**Fig. 1:**
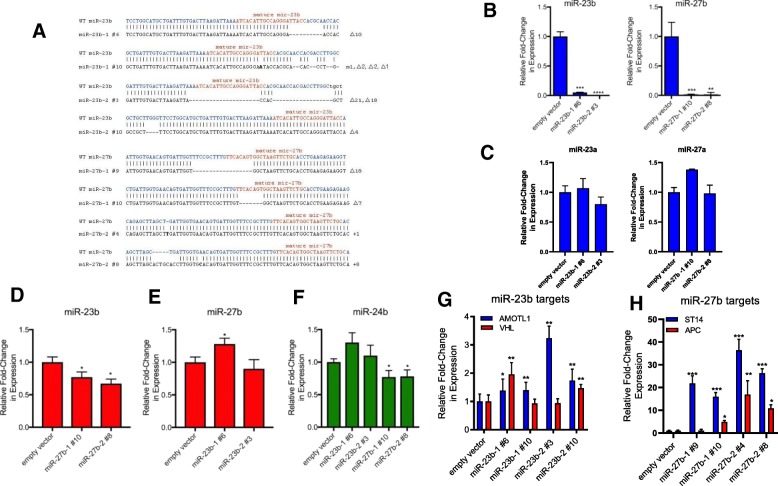
CRISPR knockout of miR-23b and miR-27b from MCF7 cells. **a**. Sequence alignment of indels present in miR-23b and miR-27b CRISPR knockout clones. **b**. qRT-PCR expression analysis of miR-23b and miR-27b in the knockout clones. **c**. qRT-PCR expression of homologs miR-23a and miR-27a in miR-23b and miR-27b knockout clones. miRNA expression is normalized to endogenous SNORD44 expression and relative to empty vector control cells, *n* = 3, ** *p* < 0.01, ***p < 0.001, ****p < 0.001, Student’s t-test. qRT-PCR of miR-23b expression in miR-27b knockout cells (**d**), miR-27b expression in miR-23b knockout cells (**e**), miR-24b expression in miR-23b and miR-27b knockout clones (**f**), normalized to SNORD44 and relative to empty vector control cells, *n* = 3, * *p* < 0.05, Student’s t-test. qRT-PCR analysis of miR-23b target genes, AMOTL1 and VHL (**g**) and miR-27b target genes ST14 and APC (**h**). Target gene expression is normalized to endogenous 36B4 expression and relative to empty vector control cells, *n* = 3, * *p* < 0.05, ** *p* < 0.01, ***p < 0.001, Student’s t-test

To investigate the functional role of miR-23b and miR-27b in breast cancer, we performed a loss-of-function study using the CRISPR/Cas9 system. We constructed CRISPR/Cas9 vectors containing 2 different sgRNAs designed to target the *MIR23B* and the *MIR27B* gene locus on chromosome 9. The sgRNAs were designed using the DNA 2.0 sgRNA design tool with the DNA sequence corresponding to the stem-loop sequence of the miRNA. Insertion of the sgRNAs into the lenti-CRISPR/Cas9 v2 vector was confirmed by DNA sequencing. MCF7 cells were transduced with the prepared lentivirus containing either empty vector or targeting sgRNAs. The T7 Endonuclease assay was used to assess the gene editing efficiency and single cell clones of each transduced cell line were plated and expanded. The genetic region surrounding the sgRNA target site of each selected clone was PCR amplified and ligated into the pCR4-TOPO TA vector and sequenced for mutation pattern determination. Indels were confirmed in single clones from each of the targeting sgRNAs (two per sgRNA), with genetic deletions observed in both miR-23b-1 and miR-23b-2 clones and deletions in both the miR-27b-1 clones, while 1 bp and 8 bp insertions were observed in the miR-27b-2 clones (Fig. 1a). Knockout of miR-23b and miR-27b was confirmed by qRT-PCR, thus demonstrating the high efficiency of the CRISPR/Cas9 system (Fig. 1b). Expression levels of the non-targeted homologs, miR-23a and miR-27a, were also measured in the knockout cells. As shown in Fig. 1c, while a slight increase in miR-27a was observed in the miR-27b-1 #10 clone, no significant changes in miR-23a or mir-27a expression were observed in any of the clones. Because miR-23b and miR-27b are located in a cluster on chromosome 9 at a distance of only 141 bp, we also assessed the expression of the non-targeted miRNA and the neighboring miR-24b in each of the clones. As shown in Fig. 1d, e the expression of the non-targeted miRNA was modestly changed in 3 out of the 4 targeted clones relative to the empty vector control. While, miR-24b was modestly increased in the miR-23b clones, and decreased in the miR-27b clones (Fig. 1f). This suggests that the presence of the indel in the adjacent miRNA precursor sequence was minimally disruptive to the normal expression of the other clustered miRNAs, relative to the highly significant repression of the targeted miRNA.

To assess the effects of miRNA knockdown, we additionally evaluated the expression of previously verified miR-23b/27b target genes. Angiomotin like 1 (AMOTL1), that promotes tube formation and migration of endothelial cells and regulates tight junctions, cell polarity, and epithelial-mesenchymal transition in epithelial cells [41], and Von Hippel-Lindau (VHL), a tumor suppressor involved in the ubiquitination and degradation of hypoxia-inducible-factor, are verified miR-23b target genes [42, 43]. Suppression of tumorigenicity 14 (ST14), also known as matriptase, is an epithelial cell-specific membrane-anchored serine protease found to reduce cell proliferation and invasion of cancer cells [23], and adenomatous polyposis coli (APC), a tumor suppressor protein that acts as an antagonist of the Wnt signaling pathway [44], are both validated miR-27b targets. In the miR-23b knockout cells, we observed a significant increase in expression of AMOTL1 in all clones tested. A significant increase in VHL levels was observed in clone miR-23b-1 #6 and miR-23b-2 #10, whereas no significant change in VHL levels was observed in 2 of the 4 clones tested (Fig. 1g). Meanwhile, in the miR-27b knockout cells, we observed a significant increase of ST14 in all clones tested, while APC expression levels were significantly elevated in 3 out of the 4 clones tested (Fig. 1h). These data indicate that knockout of miR-23b/27b affects the expression of specific target genes in breast cancer cells.

### miR-23b/27b depletion alters breast cancer cell behavior in vitro

**Fig. 2:**
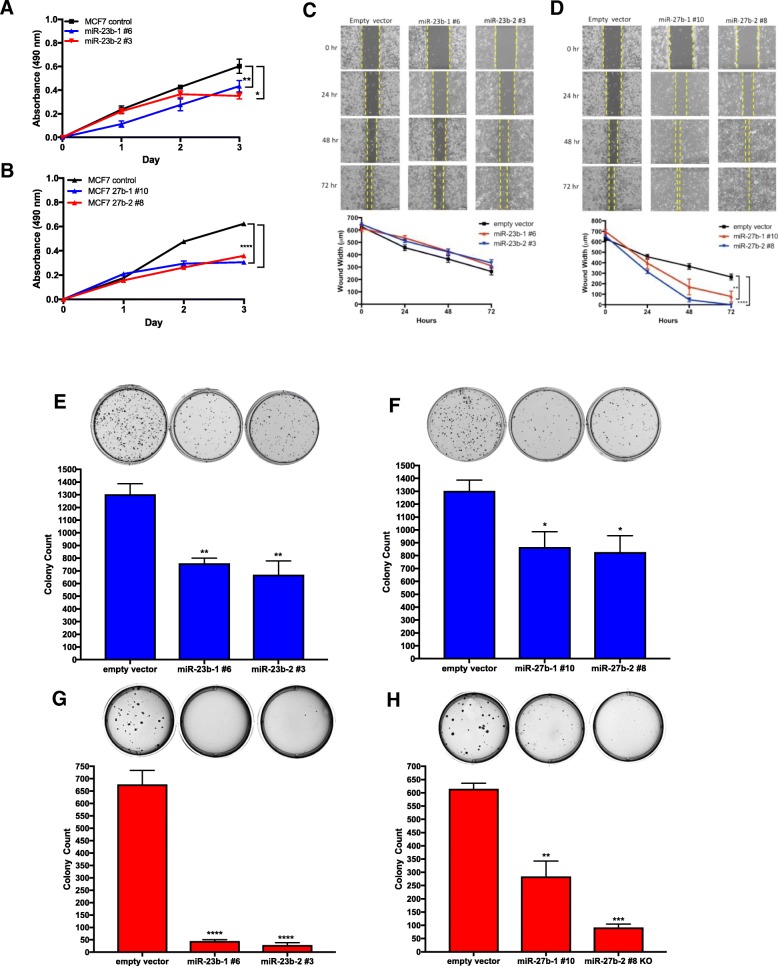
miR-23b and miR-27b knockout cell growth and migration characteristics. Cellular growth curve of miR-23b (**a**) and miR-27b (**b**) knockout clones. Cells were plated in quadruplicate and proliferation was measured by MTS assay, *p < 0.05, ** *p* < 0.01, **** *p* < 0.0001, Student’s t-test. Cell migration of miR-23b (**c**) and miR-27b (**d**) knockout cells was measured compared to empty vector control cells by the wound healing assay. A wound was made in a monolayer of cells and migration into the wound was measured every 24 h for a total of 72 h. Representative images (20X magnification) of the wound were captured (upper) and the rate of wound closure was calculated (lower); *n* = 6 per time point, ** *p* < 0.01, **** *p* < 0.0001, Two-way ANOVA. Clonogenic assay of miR-23b (**e**) and miR-27b (**f**) knockout cells compared to empty vector control cells. Soft-agar assay of miR-23b (**g**) and miR-27b (**h**) knockout cells compared to empty vector control cells. Representative images of colony growth and colony counts of the average of 2 experiments performed in triplicate; *p < 0.05, ** *p* < 0.01, ***p < 0.001, **** *p* < 0.0001, One-way ANOVA, Dunnett’s post-test

To examine the growth characteristics of the knockout cells we conducted an MTS growth assay. The rate of proliferation of both miR-23b/27b depleted cells was significantly reduced compared to the control cells (Fig. 2a, b). In addition to a reduced proliferation rate, we also assessed the migration/wound-healing rate of the knockout cells. No significant change in the rate of migration was observed in the miR-23b knockout cells compared to control cells (Fig. 2c) over a 72 h period. However, the miR-27b depleted clones migrated to close the artificial wound at a significantly faster rate compared to the control cells (Fig. 2d).

Using the clonogenic assay, we sought to assess whether the knockout cells had retained their ability to produce colonies. The knockout and control cells were plated at a low density, cultured for two weeks, stained, and counted. Both the miR-23b and miR-27b knockout clones generated significantly fewer colonies compared to control cells (Fig. 2e, f), thus demonstrating a reduced replicative ability in the absence of miR-23b/27b. We also assessed the ability of the depleted cells to grow independently of a solid surface, a known hallmark of carcinogenesis, using the soft-agar colony forming assay. Cells were suspended in agar and cultured for 20 to 30 days. The depletion of miR-23b/27b significantly attenuated anchorage-independent growth (Fig. 2g, h). These results indicated that genetic depletion of miR-23b/27b inhibits cell proliferation, colony formation, and anchorage-independent growth in vitro. However, cell migration was enhanced in miR-27b depleted cells, and unchanged in miR-23b depleted cells, indicating a functional difference between miR-23b and miR-27b, and that miR-27b plays a role in reducing cell mobility.

### miR-23b/27b knockout reduces tumor growth in vivo

**Fig. 3:**
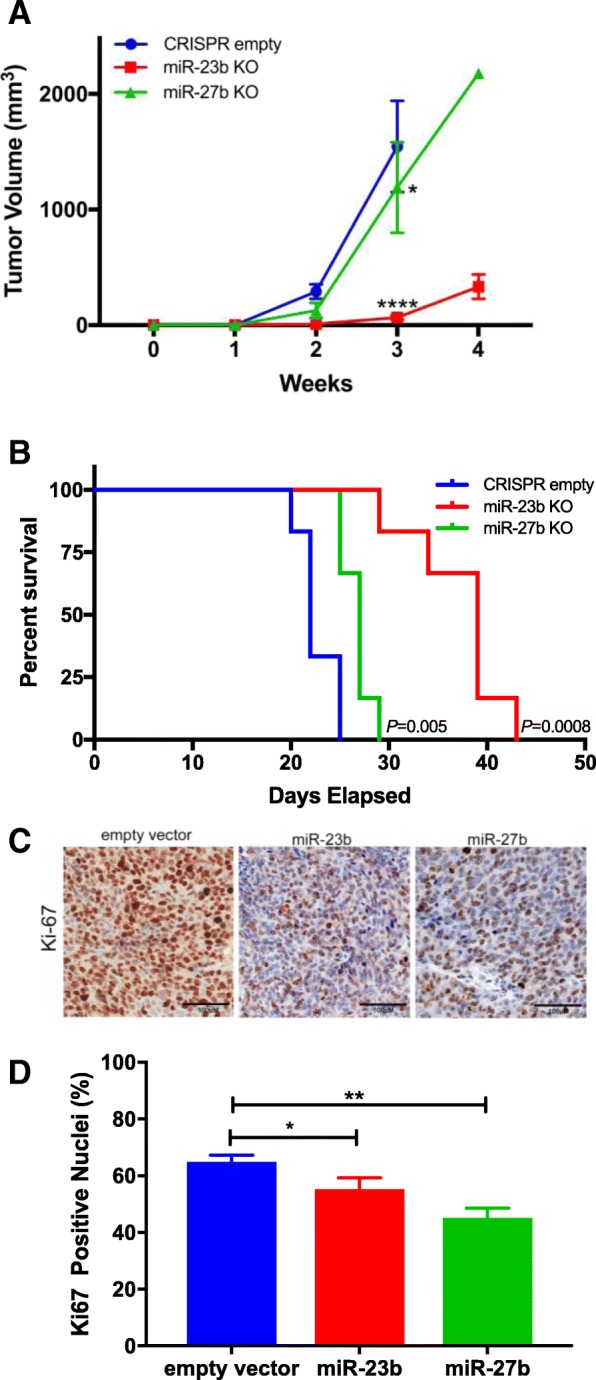
miR-23b and miR-27b knockout cell orthotopic tumor xenograft growth. **a**. Tumor xenograft growth curve, *n* = 6 mice per group, Two-way ANOVA, * *p* < 0.05, **** *p* < 0.0001. **b**. Survival analysis of empty vector, miR-23b, and miR-27b, knockout xenograft mice fed a standard diet, n = 6 mice per group. Survival curves were compared using the Log-rank (Mantel-Cox) test in GraphPad Prism. Representative images (**c**) and quantification (**d**) of Ki-67 immunohistochemistry staining of xenograft tumor tissue, black bar = 100 μm; *n* = 9 per group, *p < 0.05, ** *p* < 0.01, Student’s t-test

To evaluate the in vivo effects of genetic depletion of miR-23b/27b on tumorigenesis, we subcutaneously implanted the CRISPR empty vector control cells, miR-23b and miR-27b knockout cells (*n* = 6 mice per group, 3 groups total) into the mammary fat pad of randomly selected nude mice and monitored tumor growth for up to 6 weeks. Due to the consistent upregulation of known target genes, the miR-23b-1 #6 and the miR-27b-1 #10 clones were selected for these experiments. Due to the rapid rate of empty vector control cell xenograft growth, this group of mice was sacrificed at 3 weeks post-injection. The growth of miR-23b/miR-27b knockout xenografts was significantly reduced compared to empty vector control cells at the third week of growth as determined by tumor volumes, with the miR-23b knockout being more dramatic (Fig. 3a). Mouse survival was significantly increased in both miR-23b and miR-27b knockout xenografts (Fig. 3b). The tumor xenograft growth inhibitory effect was confirmed by immunohistochemical staining of tumor cell proliferation at the endpoint using the cell cycle marker, Ki-67 (Fig. 3c, d). These data indicate that miR-23b and miR-27b function primarily as oncogenes in breast cancer cells, and their genetic depletion significantly reduces tumor growth in vivo, with miR-23b deletion being more effective.

**Fig. 4:**
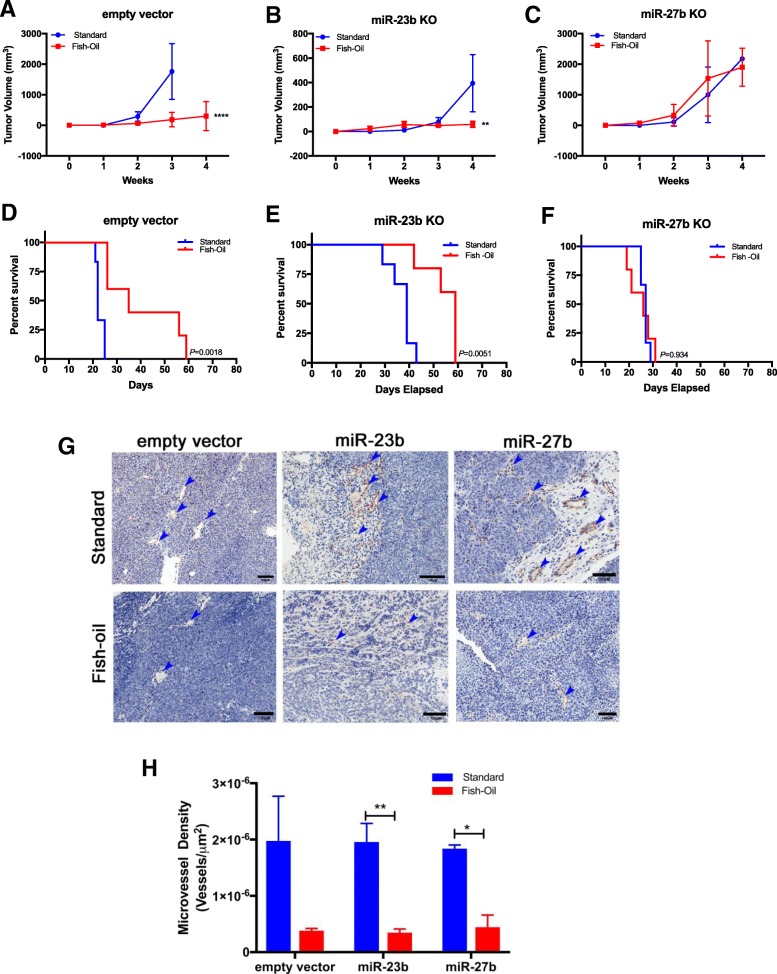
Effect of fish oil diet on the growth of miR-23b and miR-27b knockout cell orthotopic tumor xenografts. Tumor xenograft growth curve of empty vector (**a**), miR-23b (**b**) and miR-27b (**c**) knockout xenograft mice fed a fish-oil based diet versus a standard diet; n = 6 mice per group, ** *p* < 0.01, ****p < 0.0001, Two-way ANOVA. Survival analysis of empty vector (**d**), miR-23b (**e**) and miR-27b (**f**) knockout xenograft mice fed a fish-oil based diet versus a standard diet. Survival curves were compared using the Log-rank (Mantel-Cox) test in GraphPad Prism **g**. Representative images and **h**. quantitation of immunohistochemistry staining CD31 positive microvessels in xenograft tumor specimens, black bar = 100 μm; *n* = 3 per group, *p < 0.05, ** *p* < 0.01, Student’s t-test. Blue arrows indicate representative regions of CD31 positive microvessels

We previously reported that the anti-cancer and anti-angiogenesis agent docosahexaenoic acid, the primary long-chain n-3 fatty acid found in fish-oil, induced secretion of miR-23b and miR-27b via exosomes that may contribute to the anti-angiogenesis activity of DHA [32]. Therefore, we evaluated the effect of a fish-oil diet on growth and angiogenesis of MCF7 miR-23b/27b knockout xenografts. Mice were randomly selected to receive either a standard mouse diet or a fish oil-based diet consisting of 75 g/kg of fish oil, as we previously reported [45]. CRISPR empty vector control, miR-23b, and miR-27b knockout cells were subcutaneously implanted into the mammary fat pad (*n* = 6 mice per group, 3 groups total) and tumor growth and volume were monitored up to 8 weeks. The rate of growth of the empty vector and miR-23b xenografts was significantly attenuated in the mice fed a fish-oil diet, whereas the miR-27b knockout xenograft was unchanged (Fig. 4a). Survival of control and miR-23b knockout xenograft mice was significantly increased in mice fed a fish oil diet compared to those fed a standard diet, whereas the survival of miR-27b xenograft mice fed a fish oil diet was unchanged from those provided a standard diet (Fig. 4b). Immunohistochemical staining using antibodies against CD31 showed that the density of microvessels in the xenograft tumors was significantly reduced in both miR-23b and miR-27b xenograft mice fed a fish-oil diet (Fig. 4c, d). These results suggest that the anti-tumorigenic activity of DHA, provided by the fish oil diet, is abolished by miR-27b knockout in the tumor cells, while the anti-angiogenic activity of DHA is unaffected by miR-23b or miR-27b knockout.

## Discussion

Although previous studies have successfully used miRNA antagonists to reduce miRNA expression of miR-23b and miR-27b, the efficiency of knockdown is often incomplete and temporary [18]. The CRISPR/Cas9 system for gene editing and genomic depletion is significantly more efficient, producing permanent gene knockout [46]. While the majority of studies using the CRISPR/Cas9 system have focused on the depletion of protein-coding genes, very few studies have targeted non-coding RNA regions, including miRNAs for genomic depletion [47]. In this study, we utilized sgRNAs designed to target within the stem-loop sequence of miR-23b and miR-27b. The knockout efficiency was greater than 90% in all clones tested. The functional significance of miR-23b/27b depletion was examined in MCF7 breast cancer cells. Depletion of miR-23b and miR-27b allowed the re-expression of several of their target genes that are known to be involved in various mechanisms of cancer progression, indicating that expression of miR-23b and miR-27b target genes are affected by the loss of each individual miRNA.

We further characterized the growth and migratory characteristics of the miR-23b and miR-27b knockout cells in vitro. The knockdown approach, suggests that the complete elimination of expression of miR-23b and miR-27b significantly decreased the cell growth and colony forming ability of these cells compared to the empty vector control cells, strongly indicating the oncogenic nature of these two miRNAs. However, the rate of cell migration was significantly reduced in miR-27b knockout cells, while miR-23b knockout cells migratory behavior was unchanged, indicating a functional difference between these two miRNAs. When miR-23b and miR-27b knockout cell growth was measured in vivo, both tumor xenografts grew at a slower rate, with miR-23b knockout xenografts being more dramatic, compared to empty vector control, consistent with in vitro observations. However, when mice were provided a fish-oil based diet, the rate of growth of empty vector control and miR-23b knockout tumor xenografts was both significantly reduced; while the growth of miR-27b knockout xenografts was similar to that in mice fed a standard diet. Tumor neovascularization as determined by the tumor xenograft microvessel density was significantly reduced in the miR-23b and miR-27b knockout xenografts from mice fed a fish-oil based diet, regardless of the cell lines injected, which further supports the anti-angiogenic role of fish-oil and indicates that miR-23b and miR-27b are not involved in fish-oil’s anti-angiogenic activity. Some of the limitations of this study include that MCF7 cells are non-metastatic, and therefore metastasis could not be observed or evaluated in this model system; and the fish oil diet contains different n-3 polyunsaturated fatty acids and from a mechanistic point of view, a diet with DHA specific addition would be more supportive of our conclusion.

Because miR-23b and miR-27b belong to the same miRNA cluster, it is theoretically difficult to selectively and permanently eliminate the expression of one miRNA without affecting the other. In the present study, we were able to eliminate one miRNA at once with minimal effect on the other clustered miRNAs using the CRISPR/Cas9 gene editing technology, thus allowing us to dissect the functional difference of these two miRNAs. An interesting finding of the present study is that, although miR-23b and miR-27b both act primarily as oncogenic miRNAs (Figs. 2a, b, d-h and 3), miR-27b possesses context-dependent tumor suppressor activity, as evidenced by its inhibitory effect on cell migration (Fig. 2d) and the diminished effect on fish oil-induced suppression of tumor growth (Fig. 4c, f). This seems to be consistent with our in vivo data strongly indicating that miR-23b is a more potent oncogenic miRNA than miR-27b (Fig. 3a, b). Whereas the mechanisms of miR-27b’s context-dependent activity remain to be further elucidated, these observations may partially explain previous confusion over the dichotomous role of this miRNA in cancer progression.

## Conclusions

Taken together, our findings show that genetic depletion of miR-23b and miR-27b results in suppression of tumor growth, indicating that miR-23b and miR-27b are indeed oncogenic miRNAs in breast cancer. Our work suggests that the dysregulated expression of miR-23b/27b in breast cancer is a significant driving force behind breast cancer progression and therapeutic targeting of these miRNAs is a plausible strategy for breast cancer management. This study also demonstrates that miR-27b is a less potent oncogenic miRNA that also possesses context-dependent tumor suppressor activity.

## Data Availability

The datasets used and/or analyzed during the current study are available from the corresponding author on reasonable request.

## Abbreviations

CRISPR: Clustered regularly interspaced short palindromic repeats
DHA: Docosahexaenoic acid
miRNA: microRNA
sgRNA: Single-guide RNA
UTR: Untranslated region
